# Associations between biomechanical and clinical/anthropometrical factors and running-related injuries among recreational runners: a 52-week prospective cohort study

**DOI:** 10.1186/s40621-020-00237-2

**Published:** 2020-04-01

**Authors:** Jonatan Jungmalm, Rasmus Østergaard Nielsen, Pia Desai, Jon Karlsson, Tobias Hein, Stefan Grau

**Affiliations:** 1 Center for Health and Performance, Department of Food and Nutrition and Sport Science, University of Gothenburg, Box 300, SE405 30 Gothenburg, Sweden; 2 Section of Sport Science, Department of Public Health, Aarhus University, Dalgas Avenue 4, 8000 Aarhus, Denmark; 3 Department of Orthopaedics, Institute of Clinical Sciences, Sahlgrenska Academy, University of Gothenburg, Box 426, SE415 30 Gothenburg, Sweden

**Keywords:** Joint range of motion, Muscle flexibility, Muscle strength, Kinematics, Risk factors

## Abstract

**Background:**

The purpose of this exploratory study was to investigate whether runners with certain biomechanical or clinical/anthropometrical characteristics sustain more running-related injuries than runners with other biomechanical or clinical/anthropometrical characteristics.

**Methods:**

The study was designed as a prospective cohort with 52-weeks follow-up. A total of 224 injury-free, recreational runners were recruited from the Gothenburg Half Marathon and tested at baseline. The primary exposure variables were biomechanical and clinical/anthropometrical measures, including strength, lower extremity kinematics, joint range of motion, muscle flexibility, and trigger points. The primary outcome measure was any running-related injury diagnosed by a medical practitioner. Cumulative risk difference was used as measure of association. A shared frailty approach was used with legs as the unit of interest. A total of 448 legs were included in the analyses.

**Results:**

The cumulative injury incidence proportion for legs was 29.0% (95%CI = 24.0%; 34.8%). A few biomechanical and clinical/anthropometrical factors influence the number of running-related injuries sustained in recreational runners. Runners with a late timing of maximal eversion sustained 20.7% (95%CI = 1.3; 40.0) more injuries, and runners with weak abductors in relation to adductors sustained 17.3% (95%CI = 0.8; 33.7) more injuries, compared with the corresponding reference group.

**Conclusions:**

More injuries are likely to occur in runners with late timing of maximal eversion or weak hip abductors in relation to hip adductors.

## Background

Risk factors for running-related injury (RRI) have been explored in several original studies and synthesized in various systematic reviews (Ceyssens et al. 2019; Saragiotto et al. 2014; Hulme et al. 2017). Although the attempts to identify risk factors for RRI in the original articles included in these systematic reviews have been extraordinary, 86 out of 89 studies used a relative measure of association (e.g. odds ratio, relative risk, or hazard rate ratio) instead of absolute measures of association. Relative measures of association enable researchers to identify whether runners with certain characteristics are at *higher or lower risk* of RRI. For example, a recent study revealed knee stiffness as a significant predictor of injury, with an odds ratio of 1.18 (Messier et al. 2018). This reveals an 18% increased odds of sustaining RRI amongst those with high knee stiffness compared with those with low knee stiffness. Although this sounds alarming, the reader is unable to evaluate the magnitude of this value. Possibly, the 18% would be of practical importance if the odds in the low group were 0.50 and the odds in the high group were 0.59. However, it is also possible that the odds in the low group were 0.005 and the odds in the high group were 0.0059, which does not imply a clinically important difference. It is not flawed to include relative measures of association, but the limited value for practical decision-making has been highlighted and the relevance of a ratio-based result remains unclear (Nielsen et al. 2017). Moving closer to a result that is suitable for runners and their coaches, researchers within the running injury thematic also need to consider using absolute measures of association, such as cumulative risk difference (RD). RD allows the reader to assess if the potential differences between exposure groups are relevant with regard to the magnitude of the difference.

Absolute differences have been reported in biomechanical studies, although these differences are normally between injury groups and not between exposure groups (Ceyssens et al. 2019; Powers 2010). In prospective cohort studies, the most common analytical approach has been to compare differences in biomechanical measures, such as external knee adduction moments (Dudley et al. 2017), ankle eversion range of motion and eversion velocity (Kuhman et al. 2016), and hip strength (Finnoff et al. 2011), between injured and non-injured runners. Studies identifying such differences give some information on possible risk factors. However, comparing injured and non-injured subjects is not considered best practice in prospective cohort studies, as the comparison should be between exposed and non-exposed. For instance, studies can identify whether there is a difference between injured and non-injured runners in terms of knee adduction moments, but are unable to judge whether runners with a high value of knee adduction moments sustain more or less injuries compared with those runners who have a low value. Addressing the latter will assist in identifying *who* sustains most injuries and, if an absolute measure of association is used, whether or not the difference is meaningful for runners and their coaches.

In addition to that most studies use relative measures of association, a majority of studies using absolute measures of association have compared injured and non-injured runners (except a few training (Nielsen et al. 2014a; Ramskov et al. 2018a; Ramskov et al. 2018b), demographic characteristics (Nielsen et al. 2013), and foot pronation (Nielsen et al. 2014b) studies). Therefore, more studies are needed which use an absolute measure of association when examining the association between biomechanical or clinical/anthropometrical characteristics and RRI. Consequently, the purpose of the present explorative study was to investigate whether runners with certain biomechanical or clinical/anthropometrical characteristics sustain more RRI than runners with other biomechanical or clinical/anthropometrical characteristics.

## Methods

**Fig. 1:**
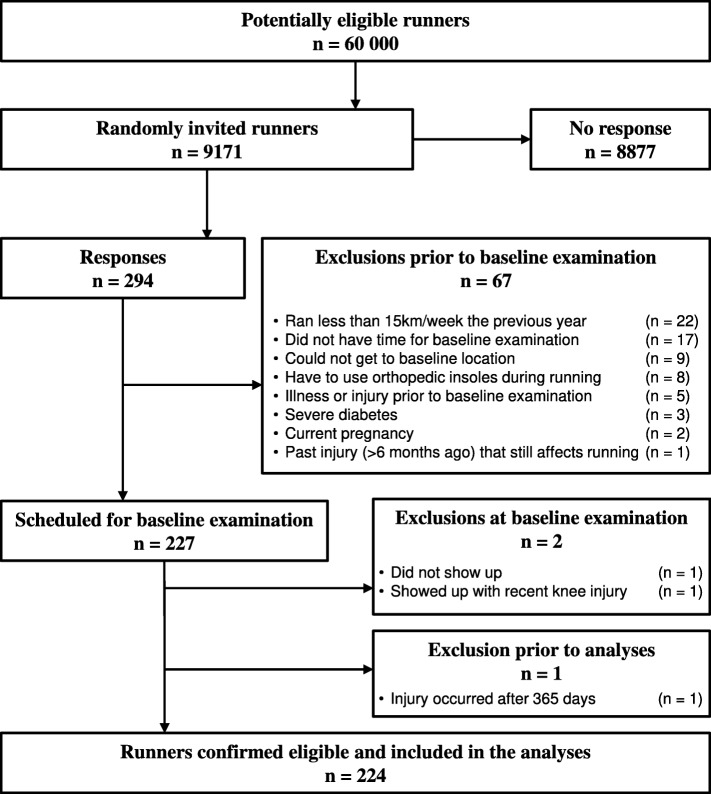
Flow chart of recruitment procedure and the inclusion and exclusion of participants. Numbers are based on participants and not legs

The study was designed as a 52-week prospective cohort study. All runners who participated in the study provided written consent prior to baseline examination. Ethical approval (DNR: 712–15) for the study and its design was obtained from the Gothenburg regional ethical review board. The study took place in Gothenburg, Sweden, and the runners were recruited from the Gothenburg Half Marathon e-mail lists provided by the Gothenburg Athletic Association. A flow chart of the recruitment procedure is presented in Fig. 1. Runners were eligible for inclusion if they did not experience any musculoskeletal injury in the lower extremities 6 months prior to baseline examination, were between the ages of 18 and 55, had an average weekly running volume of at least 15 km for the past 12 months prior to baseline examination, did not use orthopaedic insoles during running, were not pregnant, and/or did not suffer from diabetes. At baseline, participants underwent a clinical/anthropometrical examination assessing joint range of motion, muscle flexibility and trigger points, a biomechanical running analysis assessing lower extremity kinematics, and isometric strength tests. Following the baseline examination, participants were instructed to maintain their regular training habits and to report training characteristics and potential pain on a weekly basis, for a maximal period of 52 weeks. A more detailed description of the study design and its methods is published elsewhere (Jungmalm et al. 2018).

The primary outcome measure was any running-related injury diagnosed by a medical practitioner. The definition of RRI was slightly modified from the 2015 consensus statement by Yamato et al. (Yamato et al. 2015) and defined as:

*a running-related musculoskeletal pain in the lower limbs or back that causes a restriction on or stoppage of running (distance, speed, duration or training) in more than 66% of all training sessions in two consecutive weeks or in more than 50% of all training sessions in four consecutive weeks, or that requires the runner to consult a physician or other health professional.*

The modification of the original definition was adopted from a previous study (Hein et al. 2014) and due to that recreational runners do not necessarily *schedule* their training sessions in advance, which the original definition assumes.

The exposure variables were biomechanical and clinical/anthropometrical variables. Biomechanical variables consisted of lower extremity kinematics (movement) and strength measures which were measured on continuous scales. The clinical/anthropometrical variables consisted of measures of joint range of motion, muscle flexibility, and trigger points measured on categorical scales. Movement variables were hip adduction range of motion (HAD_ROM_), hip adduction velocity (HAD_VEL_), timing of maximal hip adduction (HAD_TIM_), knee flexion range of motion (KF_ROM_), maximal joint excursion knee flexion (KF_JMAX_), knee flexion velocity (KF_VEL_), timing of maximal knee flexion (KF_TIM_), rear foot eversion range of motion (REV_ROM_), rear foot eversion velocity (REV_VEL_), timing of maximal rear foot eversion (REV_TIM_), ankle dorsal flexion range of motion (ADF_ROM_), timing of maximal ankle dorsal flexion (ADF_TIM_), and ankle touch down angle (A_TD_). Movement variables were collected using a 16-camera 3D motion-capture system (Qualisys AB, Gothenburg, Sweden) with a sampling frequency of 400 Hz, while participants ran over ground at a controlled speed of 3.33 m/s (±5%) with a set of retro-reflexive markers applied according to the ISB recommendations (Wu et al. 2002). Ten strides for each side were collected and analysed through custom-written MATLAB (MathWorks Inc., Natick, MA) code, and reported as averages. Strength variables were isometric maximal voluntary strength measures (MVC) and ratios between agonistic and antagonistic muscle groups. The following strength measures were assessed: unilateral knee extension (KE_MVC_) and flexion (KF_MVC_), bilateral hip abduction (HAB_MVC_) and adduction (HAD_MVC_), left and right trunk rotation (ROT_MVC_), trunk flexion (TF_MVC_), and trunk extension (TE_MVC_). The following strength ratios were calculated: knee extension/flexion (H:Q), hip abduction/adduction (HAD:HAB), and trunk flexion/extension (TF:TE). Strength was measured using DAVID devices (David Health Solutions, Helsinki, Finland) and the maximal torque (Nm) for each test was normalized to body weight (Nm/kg).

Clinical/anthropometrical variables were measures of joint range of motion, muscle flexibility, and trigger points. Joint range of motion was assessed by the following passive measures (i) at the hip: flexion (HF_JROM_), extension (HE_JROM_), abduction (HAB_JROM_), adduction (HAD_JROM_), internal rotation (HIR_JROM_), and external rotation (HER_JROM_); (ii) at the knee: flexion (KF_JROM_), extension (KE_JROM_); and (iii) at the ankle: dorsal flexion (ADF_JROM_), plantar flexion (APF_JROM_), pronation (AP_JROM_), and supination (AS_JROM_) according to the neutral-zero method (Ryf and Weymann 1995). Muscle flexibility was assessed unilaterally for the hip flexors: rectus femoris (RF_FLEX_) and iliopsoas (IL_FLEX_) with the Thomas test, and for the hamstrings (HAM_FLEX_) with the Straight Leg Raise (Jungmalm et al. 2018). Trigger points (defined as *a tender area in a muscle that reproduces pain during palpation (*Nix 2017*)*) were assessed at the tractus iliotibialis (TI_TP_), gastrocnemius (G_TP_), soleus (S_TP_), piriformis (PF_TP_), gluteus medius (GM_TP_), tibialis posterior (TP_TP_), and tibialis anterior (TA_TP_). Participants informed the examiner whether the applied pressure at the different locations was accompanied by pain or not (Cummings and Baldry 2007).

A time-to-event analysis was conducted using days as the time-scale, with primary analysis after 365 days. Cumulative risk difference (%) was used as measure of association (Nielsen et al. 2016), calculated via the pseudo-observation method, presented with 95% confidence interval (95%CI), and considered statistically significant at *p* ≤ 0.05, and corrected for multiple testing via Bonferroni. A survival curve (Kaplan-Meier estimator) was plotted for each exposure value. Reasons for censoring (Jungmalm et al. 2019) were lack of time and/or motivation, illness, issues with training log, pregnancy, and other personal concerns hindering further participation, or RRI to the opposite leg. A shared frailty approach was used and the unit of interest was legs, meaning that each runner could have different categorizations across the two legs. Biomechanical and clinical/anthropometrical factors can be assumed to represent a characteristic of a leg; therefore legs could be used as the unit of analysis. A 68% prediction limit was used for all movement- and strength-related exposure variables, dividing each leg into one of three groups for each variable. The approach of using a 68% prediction limit has previously been discussed (Bahr and Holme 2003) but sparsely used within sports injury literature (Nilsson et al. 2012). To our knowledge, no cut-off values have been presented to differentiate biomechanical measures and to categorize participants into subgroups. Therefore, the cut-off values were defined as ±1 standard deviation (SD) from the mean, where values outside of that range were considered below or above the reference group, respectively. In terms of joint range of motion, legs outside the reference range were either assessed as hypo- or hypermobile. Only two groups were defined for exposure variables related to muscle flexibility and trigger points, since measures were dichotomized into non-restricted/restricted and no pain/pain, respectively. All analyses were performed using Stata® (StataCorp. College Station, TX).

## Results

Overall, the sample consisted of 448 legs (224 runners of which 39.6% were female). Participants had a median age of 41 years (interquartile range (IQR) =35–47), an average BMI of 22.7 kg/m^2^ (SD = ±2.1), 10 years of running experience (IQR = 5.3–17), and had covered 25 km on a weekly average the previous year (IQR = 20–39). Forty point nine percent of the sample had never experienced an RRI before. The median inclusion time (time to censoring or injury) to was 190 days (IQR = 69–361).

The cumulative injury incidence proportion for legs was 29.0% (95%CI = 24.0%; 34.8%). The total number of injured legs was 85. Of these, 38 injuries occurred only at the right side and 27 injuries occurred only at the left side, while the remaining 20 injuries were bilateral injuries occurring in 10 participants. One runner was excluded prior to the analyses, since the injury occurred at time point 367. Eventually, 448 legs were included in the analyses. Fifty legs were censored at time point 365, and 313 legs were censored prior to that according to any reason stated in the methods section. The results from the primary analyses are presented in Table 1 (biomechanical variables) and in Table 2 (clinical/anthropometrical variables), and graphically plotted in the supplementary material (Additional files 1 and 2).

**Table 1:** Biomechanical variables

	Exposure [unit]	Reference		1 standard deviation below reference			1 standard deviation above reference		
		n injuries (n total)	Risk [%]	n injuries (n total)	Risk [%] (p)	Risk difference [%] (95%CI)	n injuries (n total)	Risk [%] (p)	Risk difference [%] (95%CI)
Movement	(A) HAD_ROM_ [°]	65 (310)	32.0	12 (66)	27.7 (0.588)	−4.3 (−20.0; 11.4)	8 (66)	19.3 (0.095)	−12.7 (− 27.7; 2.2)
	(B) HAD_VEL_ [°/s]	64 (315)	31.1	13 (61)	32.6 (0.870)	1.5 (−16.1; 19.0)	7 (62)	17.9 (0.094)	−13.2 (−28.5; 2.2)
	(C) HAD_TIM_ [%]	64 (334)	29.7	10 (48)	32.2 (0.780)	2.5 (−14.9; 19.8)	11 (60)	25.6 (0.624)	−4.1 (−20.7; 14.4)
	(D) KF_ROM_ [°]	62 (343)	28.2	12 (48)	30.5 (0.796)	−2.3 (−15.5; 20.1)	11 (51)	36.6 (0.401)	8.4 (−11.2; 27.9)
	(E) KF_JMAX_ [°]	58 (302)	28.8	13 (74)	24.3 (0.541)	−4.5 (−19.0; 9.9)	14 (66)	38.1 (0.236)	9.3 (−6.1; 24.7)
	(F) KF_VEL_ [°/s]	56 (297)	29.5	14 (68)	27.2 (0.783)	−2.3 (−18.4; 13.9)	14 (71)	32.8 (0.675)	3.3 (−12.2; 18.9)
	(G) KF_TIM_ [%]	68 (343)	30.9	5 (37)	18.1 (0.140)	−12.8 (−29.9; 4.2)	12 (62)	27.8 (0.702)	−3.1 (− 19.2; 12.9)
	(H) REV_ROM_ [°]	63 (309)	30.8	10 (61)	21.5 (0.272)	−9.3 (−26.0; 7.3)	12 (72)	30.2 (0.930)	−0.6 (−15.0; 13.7)
	(J) REV_VEL_ [°/s]	58 (303)	28.8	13 (66)	28.6 (0.981)	−0.2 (−16.9; 17.3)	13 (67)	34.2 (0.487)	5.4 (−9.8; 20.6)
	(K) REV_TIM_ [%]	55 (316)	25.7	9 (52)	28.1 (0.786)	2.4 (−15.2; 20.0)	21 (74)	46.4 (0.033)*	20.7 (1.3; 40.0)
	(L) ADF_ROM_ [°]	60 (333)	27.3	10 (48)	31.8 (0.610)	4.5 (−12.7; 21.6)	15 (61)	38.9 (0.193)	11.6 (−5.9; 29.1)
	(M) A_TD_ [°]	58 (319)	28.5	12 (66)	24.7 (0.650)	−3.8 (−20.2; 12.6)	15 (57)	40.3 (0.186)	11.8 (−5.7; 29.4)
	(N) ADF_TIM_ [%]	72 (355)	30.6	4 (31)	17.0 (0.115)	−14.6 (−32.9; 3.7)	9 (56)	22.3 (0.244)	−9.3 (−24.9; 6.3)
Strength [Nm/kg]	(O) HAB_MVC_	64 (310)	30.5	11 (66)	28.1 (0.793)	−2.4 (− 20.2; 15.5)	10 (72)	23.3 (0.332)	−7.2 (− 21.7; 7.3)
	(P) HAD_MVC_	58 (306)	29.4	16 (72)	34.0 (0.634)	4.6 (−14.5; 23.8)	11 (70)	21.8 (0.328)	−7.6 (−22.8; 7.6)
	(Q) KE_MVC_	50 (297)	25.3	22 (77)	42.8 (0.065)	17.5 (−1.0; 36.1)	13 (74)	29.9 (0.590)	4.6 (−12.1; 21.4)
	(R) KF_MVC_	61 (322)	29.0	12 (70)	26.2 (0.741)	−2.8 (−19.7; 14.0)	12 (56)	32.8 (0.645)	3.8 (−12.5; 20.2)
	(S) ROT_MVC_	62 (311)	31.7	9 (66)	15.7 (0.065)	−16.0 (−33.1; 1.0)	12 (65)	27.3 (0.600)	−4.4 (−20.6; 11.9)
	(T) TF_MVC_	52 (300)	27.4	11 (70)	23.0 (0.608)	−4.4 (− 21.4; 12.5)	22 (78)	40.9 (0.118)	13.5 (−3.4; 30.3)
	(U) TE_MVC_	60 (316)	29.7	14 (66)	30.6 (0.925)	0.9 (−18.9; 17.2)	10 (62)	26.0 (0.678)	−3.7 (−21.3; 13.9)
	(V) HAB:HAD	57 (320)	26.3	17 (62)	43.6 (0.040)*	17.3 (0.8; 33.7)	11 (66)	28.7 (0.774)	2.4 (−14.0; 18.8)
	(X) H:Q	65 (332)	27.8	7 (59)	24.8 (0.666)	−3.0 (−16.8; 10.7)	13 (57)	40.7 (0.156)	12.9 (−4.9; 30.8)
	(Y) TF:TE	61 (312)	29.8	11 (70)	23.2 (0.436)	−6.6 (−23.0; 9.9)	12 (62)	31.6 (0.820)	1.8 (−13.7; 17.3)

*HAD*_*ROM*_ Hip adduction range of motion, *HAD*_*VEL*_ Hip adduction velocity, *HAD*_*TIM*_ Timing of maximal hip adduction, *KF*_*ROM*_ Knee flexion range of motion, *KF*_*JMAX*_ Maximal joint excursion knee flexion, *KF*_*VEL*_ Knee flexion velocity, *KF*_*TIM*_ Timing of maximal knee flexion, *REV*_*ROM*_ Rear foot eversion range of motion, *REV*_*VEL*_ Rear foot eversion velocity, *REV*_*TIM*_ Timing of maximal rear foot eversion, *ADF*_*ROM*_ Ankle dorsal flexion range of motion, *A*_*TD*_ Ankle touch down angle, *ADF*_*TIM*_ Timing of maximal ankle dorsal flexion, *HAB*_*MVC*_ Hip abduction strength, *HAD*_*MVC*_ Hip adduction strength, *KE*_*MVC*_ Knee extension strength, *KF*_*MVC*_ Knee flexion strength, *ROT*_*MVC*_ Trunk rotation strength, *TF*_*MVC*_ Trunk flexion strength, *TE*_*MVC*_ Trunk extension strength, *HAB:HAD* Hip abduction:adduction strength ratio, *H:Q* Hamstring:quadriceps strength ratio (knee flexion:extension), *TF:TE* Trunk flexion:extension strength ratio. * indicates *p* ≤ 0.05. Letters in parentheses (A-Y) indicate a corresponding Kaplan-Meier plot presented in the supplementary material (see Additional file 1)

**Table 2:** Clinical/anthropometrical variables

	Exposure	Reference		Hypermobile			Hypomobile		
		n injuries (n total)	Risk [%]	n injuries (n total)	Risk [%] (p)	Risk difference [%] (95%CI)	n injuries (n total)	Risk [%] (p)	Risk difference [%] (95%CI)
Joint range of motion	(A) HF_JROM_	77 (419)	28.1	0 (0)	NA	NA	8 (29)	41.3 (0.286)	13.2 (−11.1; 37.5)
	(B) HE_JROM_	83 (437)	29.2	2 (6)	44.4 (0.569)	15.2 (−37.0; 67.4)	0 (5)	0	NA
	(C) HAB_JROM_	80 (420)	29.5	2 (12)	24.6 (0.822)	−4.9 (− 47.4; 37.6)	3 (16)	20.2 (0.539)	−9.3 (−39.0; 20.4)
	(D) HAD_JROM_	85 (442)	29.4	0 (1)	0	NA	0 (5)	0	NA
	(E) HIR_JROM_	85 (442)	27.8	0 (1)	0	NA	0 (5)	0	NA
	(F) HER_JROM_	63 (341)	28.5	17 (91)	27.4 (0.855)	−1.1 (−15.7; 13.5)	5 (16)	49.6 (0.166)	21.1 (−8.7; 50.8)
	(G) KF_JROM_	82 (436)	28.8	1 (4)	33.8 (0.896)	5.0 (−69.5; 79.4)	2 (8)	35.8 (0.724)	7.0 (−31.9; 45.9)
	(H) KE_JROM_	52 (284)	26.5	27 (122)	36.9 (0.160)	10.4 (−4.1; 24.9)	6 (42)	22.9 (0.680)	−3.6 (−20.7; 13.5)
	(J) DF_JROM_	67 (346)	28.6	0 (2)	0	NA	18 (100)	31.3 (0.682)	2.7 (−10.2; 15.6)
	(K) PF_JROM_	85 (448)	29.0	0 (0)	NA	NA	0 (0)	NA	NA
	(L) AP_JROM_	71 (379)	28.7	9 (43)	36.2 (0.525)	7.5 (−15.7; 30.8)	5 (26)	22.2 (0.610)	−6.5 (−31.3; 18.4)
	(M) AS_JROM_	59 (312)	27.8	16 (94)	31.8 (0.549)	4.0 (−9.0; 17.0)	10 (42)	31.9 (0.730)	4.1 (−19.2; 27.4)
		Reference		Restricted (MF) / Pain (TP)					
		n injuries (n total)	Risk [%]	n injuries (n total)		Risk [%] (p)		Risk difference [%] (95%CI)	
Muscle flexibility	(N) RF_FLEX_	42 (231)	29.8	43 (217)		28.6 (0.845)		−1.2 (−13.2; 10.8)	
	(O) IL_FLEX_	43 (212)	31.5	42 (236)		27.0 (0.465)		−4.5 (−16.4; 7.5)	
	(P) HAM_FLEX_	30 (165)	29.7	55 (283)		29.1 (0.992)		−0.1 (−12.3; 12.1)	
Trigger points	(Q) TI_TP_	42 (228)	29.1	43 (220)		29.4 (0.966)		0.3 (−11.5; 12.0)	
	(R) G_TP_	24 (167)	24.8	61 (281)		31.8 (0.288)		7.0 (−4.3; 18.4)	
	(S) S_TP_	46 (295)	25.6	39 (153)		36.2 (0.109)		10.6 (−2.4; 23.5)	
	(T) PF_TP_	59 (330)	28.6	26 (118)		31.0 (0.743)		2.4 (−12.0; 16.8)	
	(U) GM_TP_	54 (298)	27.9	31 (150)		31.7 (0.549)		3.8 (−8.6; 16.2)	
	(V) TP_TP_	58 (316)	28.1	27 (132)		31.9 (0.604)		3.8 (−10.4; 18.0)	
	(X) TA_TP_	71 (391)	27.7	14 (57)		39.2 (0.167)		11.5 (−4.8; 27.9)	

*HF*_*JROM*_ Hip flexion joint range of motion, *HE*_*JROM*_ Hip extension joint range of motion, *HAB*_*JROM*_ Hip abduction joint range of motion, *HAD*_*JROM*_ Hip adduction joint range of motion, *HIR*_*JROM*_ Hip internal rotation joint range of motion, *HER*_*JROM*_ Hip external rotation joint range of motion, *KF*_*JROM*_ Knee flexion joint range of motion, *KE*_*JROM*_ Knee extension joint range of motion, *DF*_*JROM*_ Ankle dorsal flexion joint range of motion, *PF*_*JROM*_ Ankle plantar flexion joint range of motion, *AP*_*JROM*_ Ankle pronation joint range of motion, *AS*_*JROM*_ Ankle supination joint range of motion, *RF*_*FLEX*_ Rectus femoris flexibility, *IL*_*FLEX*_ Iliopsoas flexibility, *HAM*_*FLEX*_ Hamstrings flexibility, *TI*_*TP*_ Tractus iliotibialis trigger point, *G*_*TP*_ Gastrocnemius trigger point, *S*_*TP*_ Soleus trigger point, *PF*_*TP*_ Piriformis trigger point, *GM*_*TP*_ Gluteus medius trigger point, *TP*_*TP*_ Tibialis posterior trigger point, *TA*_*TP*_ Tibialis anterior trigger point, *MF* Muscle Flexibility, *NA* Not applicable. Letters in parentheses (A-X) indicate a corresponding Kaplan-Meier plot presented in the supplementary material [see Additional file 2]

For movement variables, the only statistically significant characteristic found was timing of maximal eversion (REV_TIM_), where runners in the +1SD-group (having relatively late timing) sustained more injuries than the reference group (RD = 20.7, 95%CI = 1.3; 40.0). For strength variables, the only statistically significant characteristic found was a low HAB:HAD ratio. Runners with weak abductors in relation to the adductors sustained more RRI compared with runners in the reference group (RD = 17.3, 95%CI = 0.8; 33.7).

Measures from joint range of motion, muscle flexibility and trigger point tests did not reveal any significant differences between exposure groups. Consequently, the data from this study suggest no associations at all between excessive or restricted joint range of motion, excessive or restricted muscle flexibility or having painful trigger points, and RRI.

## Discussion

The purpose of this study was to exploratively investigate whether runners with certain biomechanical or clinical/anthropometrical characteristics sustain more RRI than runners having other biomechanical or clinical/anthropometrical characteristics. Overall, the cumulative incidence proportion of RRI reported in this study appears to be comparable with what previous studies have reported (Yamato et al. 2015). However, it is important to highlight that this study reported the incidence proportion for injured *legs* and not for injured *participants*.

The finding of the association between a relatively late timing of ankle eversion and RRI might be an indication of insufficient neuromuscular activation of tibialis posterior, since this muscle work eccentrically during the pronation movement. However, the evidence on the association between lower-leg kinematics, specifically ankle eversion, and RRI is conflicting (Ceyssens et al. 2019; Munteanu and Barton 2011) and must be taken with caution. Runners having low eversion ROM (REV_ROM_) and runners with high eversion velocity (REV_VEL_) seem to sustain more injuries than runners within the corresponding reference groups. Taken together, runners, clinicians, and coaches should be aware of, and pay extra attention to subgroups of runners with certain movement characteristics – such as runners with a relatively late timing of ankle eversion – as they may be more susceptible to RRI.

In addition to runners having a low HAB:HAD ratio, runners with low knee extension strength (KE_MVC_) showed a tendency towards sustaining more injuries than runners in the reference group (RD = 17.5, 95%CI = -1.0; 36.1). Weak hip abductors and weak knee extensors have previously been discussed as key factors in the development of RRI, especially hip and knee injuries (Powers 2010). The present study supports that runners with weak hip abductors and potentially runners with weak knee extensors sustain more injuries than those with relatively greater strength.

Runners with decreased mobility for hip external rotation and increased mobility for knee extension showed a tendency to sustain more injuries than runners in the corresponding reference group. A reason for the uncertainty regarding hip external rotation is that only 16 legs were categorized into the decreased mobility-group, and that only five injuries occurred within that group.

None of the seven measures for trigger points appear to suggest that runners with painful trigger points sustain significantly more RRI compared with runners without painful trigger points. However, we cannot completely disregard trigger points in future studies since it seems like runners with painful trigger points in G_TP_, S_TP_ or TA_TP_, (i.e. trigger points apparent in Gastrocnemius, Soleus or Tibialis Anterior) sustained more injuries than runners without painful trigger points. Due to the subjective experience of pain, persons with lower pain tolerance may have a higher tendency to report injuries whereas others with higher pain tolerance may do the opposite. A clear definition of RRI is therefore necessary to increase the unanimity between researchers and runners of what an injury is, regardless of level of pain tolerance. In future studies, measuring the actual structural damage would likely be even better. Another possible reason behind this might be that a painful trigger point can be an early sign of a structure exposed to a higher training load than it is prepared for. Since an injury occurs when the load exceeds the load-capacity of a specific structure (Hreljac 2005), runners with painful trigger points might be closer to sustaining an injury. To the authors’ knowledge, the association between trigger points and RRI among recreational runners has not been investigated in previous literature.

The major strength of the present study was the comprehensive baseline screening and the prospective study design. Moreover, the procedure for diagnosing RRI was superior to the commonly-used self-report approach. A limitation of the study was the low number of injuries, which did not reach ten for each exposure variable. Further, this study did not have enough power to analyse specific RRIs in recreational runners. More participants (and more injuries) might have allowed for competing-risk analyses, which would have been a beneficial statistical tool to target the question of what type of runners sustain more or less specific RRIs. Competing-risk analyses would also enable the examination of patterns for different types of injuries. It is plausible that some exposure variables show conflicting associations with RRI when all injuries are grouped together. Moreover, as we only measured each runner once, one limitation of this study is that we were unable to quantify changes from baseline that possibly occurred prior to injury. A final limitation includes the difficulty to infer specific RRI based on running biomechanics, as shown in a recent study (Jauhiainen et al. 2020), might not be solved by incorporating clinical/anthropometrical measures, but could be an important step. Sources of potential bias in the present study could be the use of days as the time scale, since runners were only able to sustain an injury on a day with a running session. However, they were considered at risk of sustaining injury all 365 days. Another potential bias might be the runners’ motivation to submit training and injury information determined which runners continued through follow-up, since a major reason for censoring was lack of time and/or motivation (33.6%). Other reasons for censoring were injury to opposite leg (17.9%), illness/injury not related to running (8.3%), issues with training log (0.6%) and completing follow-up (39.7%). Importantly, the findings from this study can only be generalised to similar runners, which are adult, healthy Swedish recreational runners.

## Conclusion

The results from the present study add new information regarding whether runners with certain characteristics sustain more RRI than runners with other characteristics. More injuries are likely to occur in runners with late timing of maximal eversion or weak abductors in relation to adductors. Importantly, the present study report *who* sustain more running-related injuries than others, but is unable to eventually inform *why* certain runners sustain more injuries than others (as the study did not investigate injury mechanisms). Coaches and athletes can use this information in the planning of the (running) training, for instance to if they are more likely to committing a training error that can explain why they sustain injury.

## Supplementary information

**Additional file 1.**

**Additional file 2.**

## Data Availability

A pseudonymised dataset used in the current study are available from the corresponding author on reasonable request.

## Abbreviations

95%CI: 95% Confidence interval
AP_JROM_: Ankle pronation joint range of motion
AS_JROM_: Ankle supination joint range of motion
ADF_ROM_: Ankle dorsal flexion range of motion
A_TD_: Ankle touch down angle
ADF_TIM_: Timing of maximal ankle dorsal flexion
BMI: Body mass index
DF_JROM_: Ankle dorsal flexion joint range of motion
GM_TP_: Gluteus medius trigger point
G_TP_: Gastrocnemius trigger point
HAD_ROM_: Hip adduction range of motion
HAD_VEL_: Hip adduction velocity
HAD_TIM_: Timing of maximal hip adduction
HAB_MVC_: Hip abduction strength
HAD_MVC_: Hip adduction strength
HAB:HAD: Hip abduction:adduction strength ratio
H:Q: Hamstring:quadriceps strength ratio
HF_JROM_: Hip flexion joint range of motion
HE_JROM_: Hip extension joint range of motion
HAB_JROM_: Hip abduction joint range of motion
HAD_JROM_: Hip adduction joint range of motion
HIR_JROM_: Hip internal rotation joint range of motion
HER_JROM_: Hip external rotation joint range of motion
HAM_FLEX_: Hamstrings flexibility
IL_FLEX_: Iliopsoas flexibility
ISB: International society of biomechanics
IQR: Interquartile range
KF_ROM_: Knee flexion range of motion
KF_JMAX_: Maximal joint excursion knee flexion
KF_VEL_: Knee flexion velocity
KF_TIM_: Timing of maximal knee flexion
KE_MVC_: Knee extension strength
KF_MVC_: Knee flexion strength
KF_JROM_: Knee flexion joint range of motion
KE_JROM_: Knee extension joint range of motion
MF: Muscle Flexibility
NA: Not applicable
PF_JROM_: Ankle plantar flexion joint range of motion
PF_TP_: Piriformis trigger point
RRI: Running-related injury
RD: Risk difference
REV_ROM_: Rear foot eversion range of motion
REV_VEL_: Rear foot eversion velocity
REV_TIM_: Timing of maximal rear foot eversion
ROT_MVC_: Trunk rotation strength
RF_FLEX_: Rectus femoris flexibility
SD: Standard deviation
S_TP_: Soleus trigger point
TF_MVC_: Trunk flexion strength
TE_MVC_: Trunk extension strength
TF:TE: Trunk flexion:extension strength ratio
TI_TP_: Tractus iliotibialis trigger point
TP_TP_: Tibialis posterior trigger point
TA_TP_: Tibialis anterior trigger point
