# Supplementary material for: Associations between biomechanical and clinical/anthropometrical factors and running-related injuries among recreational runners: a 52-week prospective cohort study
Source: Inj Epidemiol. 2020 Apr 1;7:10. doi: 10.1186/s40621-020-00237-2 (PMC7110719; doi:10.1186/s40621-020-00237-2)
Supplement: Supplementary file 1 — Additional file 1. [file 40621_2020_237_MOESM1_ESM.pdf]

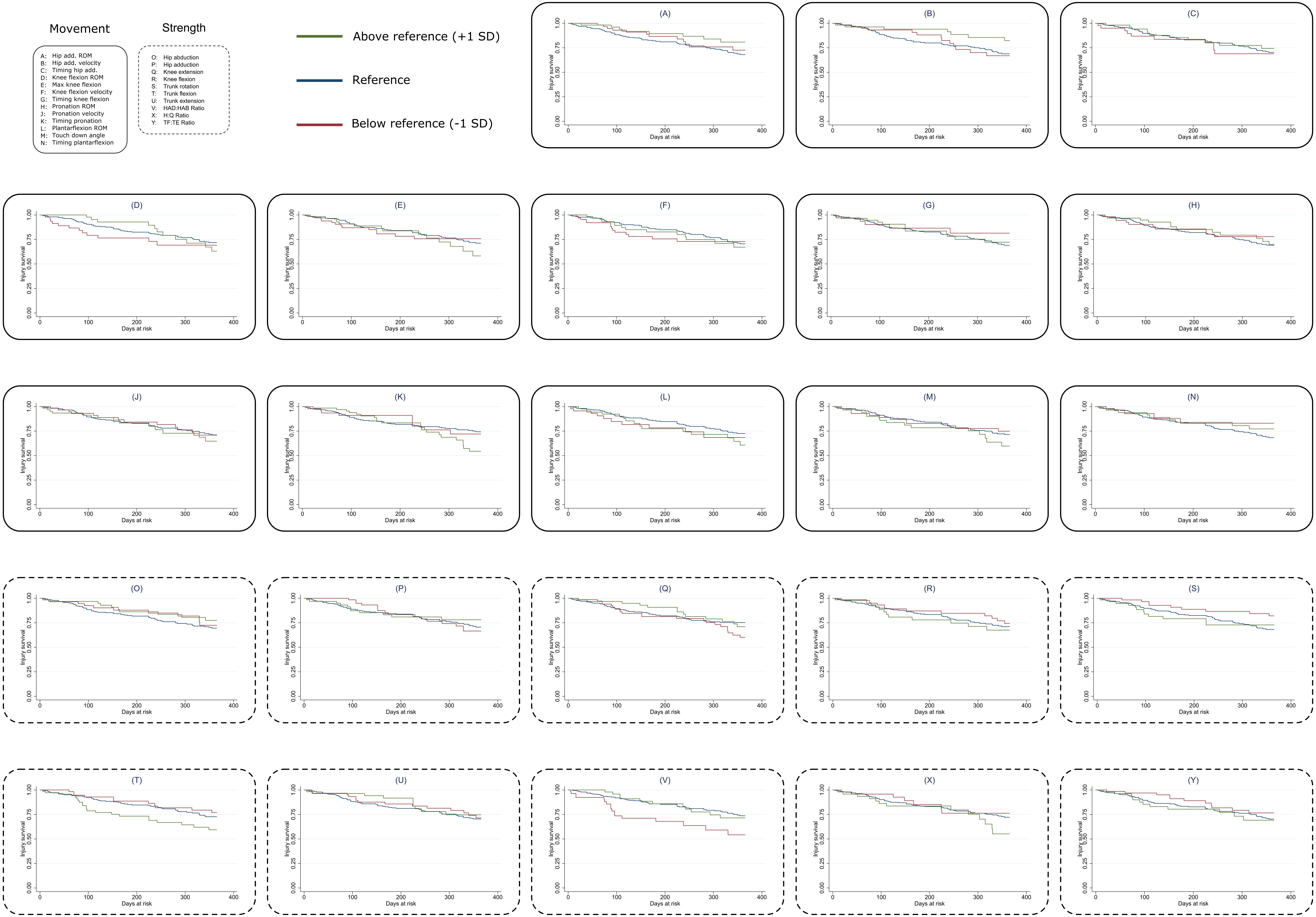
Movement
A: Hip add. ROM
B: Hip add. velocity
C: Timing hip add.
D: Knee flexion ROM
E: Max knee flexion
F: Knee flexion velocity
G: Timing knee flexion
H: Pronation ROM
J: Pronation velocity
K: Timing pronation
L: Plantarflexion ROM
M: Touch down angle
N: Timing plantarflexion
Strength
O: Hip abduction
P: Hip adduction
Q: Knee extension
R: Knee flexion
S: Trunk rotation
T: Trunk flexion
U: Trunk extension
V: HAD:HAB Ratio
X: H:Q Ratio
Y: TF:TE Ratio
Above reference (+1 SD)
Reference
Below reference (-1 SD)
(A)
(B)
(C)
(D)
(E)
(F)
(G)
(H)
(J)
(K)
(L)
(M)
(N)
(O)
(P)
(Q)
(R)
(S)
(T)
(U)
(V)
(X)
(Y)
Injury survival
Days at risk
