# Supplementary material for: Associations between biomechanical and clinical/anthropometrical factors and running-related injuries among recreational runners: a 52-week prospective cohort study
Source: Inj Epidemiol. 2020 Apr 1;7:10. doi: 10.1186/s40621-020-00237-2 (PMC7110719; doi:10.1186/s40621-020-00237-2)
Supplement: Supplementary file 2 — Additional file 2. [file 40621_2020_237_MOESM2_ESM.pdf]

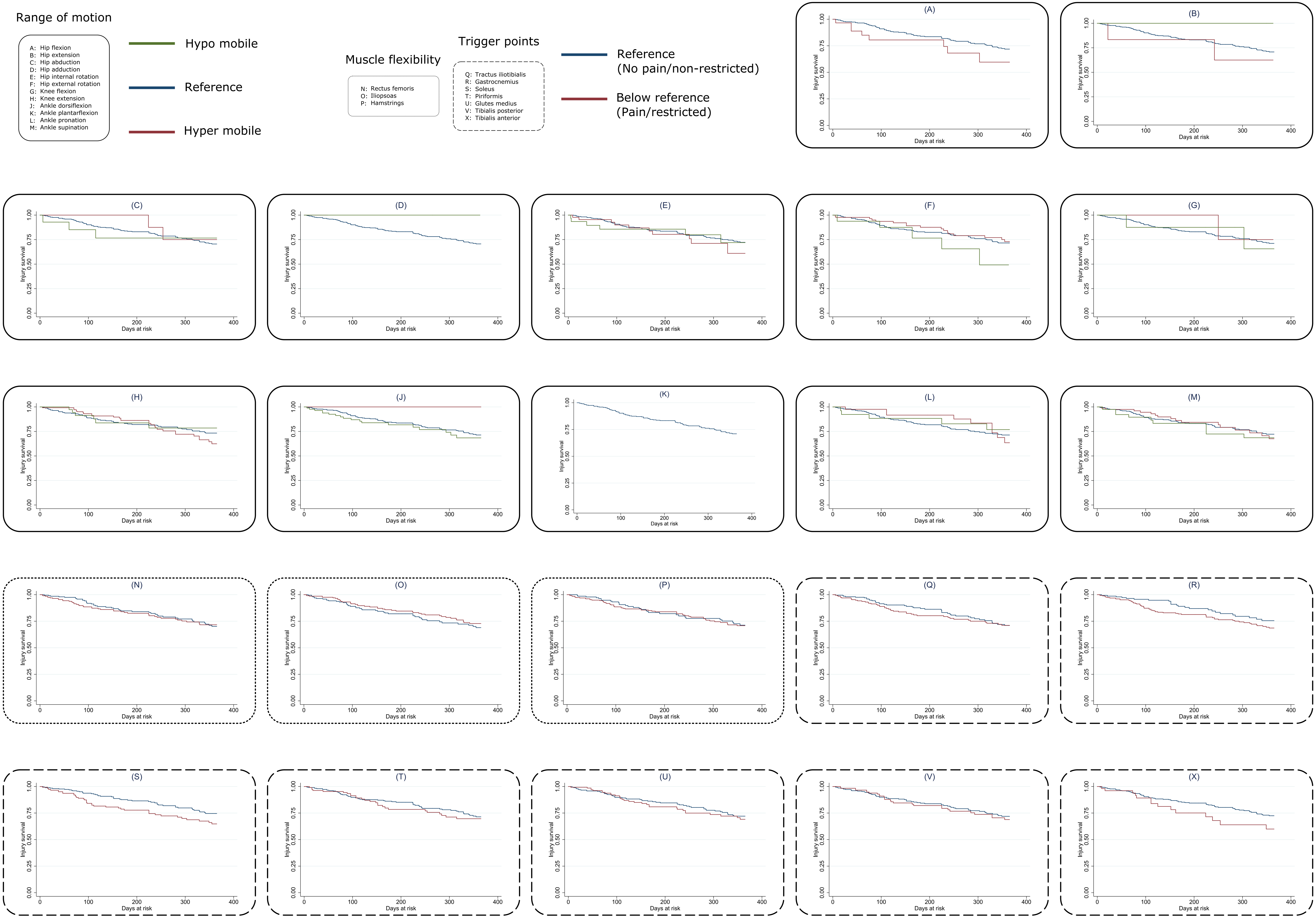
Range of motion
A: Hip flexion
B: Hip extension
C: Hip abduction
D: Hip adduction
E: Hip internal rotation
F: Hip external rotation
G: Knee flexion
H: Knee extension
J: Ankle dorsiflexion
K: Ankle plantarflexion
L: Ankle pronation
M: Ankle supination
Hypo mobile
Reference
Hyper mobile
Muscle flexibility
N: Rectus femoris
O: Iliopsoas
P: Hamstrings
Trigger points
Q: Tractus iliotibialis
R: Gastrocnemius
S: Soleus
T: Piriformis
U: Glutes medius
V: Tibialis posterior
X: Tibialis anterior
Reference
(No pain/non-restricted)
Below reference
(Pain/restricted)
(A)
(B)
(C)
(D)
(E)
(F)
(G)
(H)
(J)
(K)
(L)
(M)
(N)
(O)
(P)
(Q)
(R)
(S)
(T)
(U)
(V)
(X)
Injury survival
Days at risk
0.00
0.25
0.50
0.75
1.00
0
100
200
300
400
